# Metastatic Anorectal Melanoma Presenting as Seizures: An Infrequent Culprit

**DOI:** 10.3390/diseases10020021

**Published:** 2022-04-08

**Authors:** Joao Galante, Sola Adeleke, Rosemeen Parkar, Nipin Bagla, Albert Edwards, Stergios Boussios, Rakesh Raman

**Affiliations:** 1 Maidstone Hospital, Hermitage Lane, Maidstone ME16 9QQ, UK; joao.galante@nhs.net (J.G.); rosemeen.parkar@nhs.net (R.P.); nipin.bagla@nhs.net (N.B.); albert.edwards@nhs.net (A.E.); rakeshraman@nhs.net (R.R.); 2 High Dimensional Neurology Group, UCL Queen’s Square Institute of Neurology, London WC1N 3BG, UK; sola.adeleke@nhs.net; 3 Department of Oncology, Guy’s and St Thomas’ Hospital, London SE1 7EH, UK; 4 School of Cancer & Pharmaceutical Sciences, King’s College London, Strand, London WC2R 2LS, UK; 5 Department of Medical Oncology, Medway NHS Foundation Trust, Windmill Road, Gillingham ME7 5NY, UK; 6 School of Cancer & Pharmaceutical Sciences, Faculty of Life Sciences & Medicine, King’s College London, London SE1 9RT, UK; 7 AELIA Organization, 9th Km Thessaloniki—Thermi, 57001 Thessaloniki, Greece

**Keywords:** melanoma, anorectal, malignant, metastatic, seizures, diagnostic, pathways

## Abstract

Anorectal malignant melanoma is a rare culprit of malignancies in the anorectal region. With a presentation that mimics the vastly more common colorectal tumours, clinical misdiagnosis and diagnostic delays often occur, contributing to a dismal prognosis. The authors report a case of metastatic anorectal malignant melanoma presenting as seizures. Though our standard diagnostic pathway for suspected anorectal malignancies was followed, and despite the patient having computerized tomography (CT) of the head earlier, this presentation nonetheless led to a prolongation of time needed to reach histological diagnosis and delay in commencing definitive treatment. It also highlights the paucity of research into the pathophysiology and management of this infrequent but aggressive disease, and the need for raising awareness about this condition to the medical community so that it is considered as a plausible differential diagnosis from the outset and diagnostic pathways adjusted accordingly.

## 1. Introduction

Anorectal malignant melanoma (ARMM) is rare, accounting for less than 2% of all melanomas [1], and between 0.05% to 5% of all anorectal malignancies. It is most commonly found in women over the age of 50 [2,3]. ARMM tends to arise from the melanocytes found near the dentate line [4]. Embryologically, the anorectum arises at the junction between the endoderm and ectoderm, making this a potentially unstable location that is prone to aberrant cellular differentiation and proliferation [4,5]. Furthermore, lesions above the dentate line may be at increased risk of developing distant metastasis due to their drainage into the portal circulation [6]. Anorectal melanomas tend to present with the symptoms of classic anorectal neoplasms such as bleeding, tenesmus, abdominal pain, constipation, and decreased stool calibre [7]. As this is an uncommon location for melanoma, it will initially follow the typical diagnostic pathway for anorectal masses including digital rectal examination, proctoscopy/sigmoidoscopy/colonoscopy, and staging with high resolution magnetic resonance imaging (MRI) of the pelvis and computerized tomography (CT) of the chest, abdomen, and pelvis (CT-CAP), and it is not until biopsy results have been returned that melanoma diagnosis can be confirmed.

Hence, clinical misdiagnosis has been reported in nearly 60% of cases [8,9]. At the time of presentation, over 70% of patients may have metastatic disease [10].

There is paucity of data on the management of metastatic ARMM. Therapeutic approaches for metastatic disease are mostly based on standard systemic therapy for metastatic (cutaneous) melanoma. Prior to the era of immunotherapy, 5-year survival for ARMM was reported to be between 6% to 22%, and there is a median survival time of 12.2 to 22 months [2]. Here, we describe the challenging diagnostic course of a case of ARMM with an atypical metastatic presentation.

## 2. Case Description

A 69-year-old was brought to the emergency department following a witnessed first seizure, described as 2–3 min of generalized tonic–clonic activity, pallor, and sweating, with tongue biting and urinary incontinence, followed by a postictal state.

He had history of hypertension and clinical hypothyroidism, controlled by amlodipine and levothyroxine respectively. There was a long history of consuming more than 50 units of alcohol per week before this presentation, which had been reduced to 14–28 units by the time of presentation.

Physical examination demonstrated mild bilateral hand tremor and bilateral pitting oedema to mid-shin level. He was clinically euthyroid. A 12-lead electrocardiogram showed sinus tachycardia, and a plain chest radiograph was unremarkable. Full blood count, renal function, liver function, electrolytes, and C-reactive protein (CRP) were within normal range.

**Figure 1:**
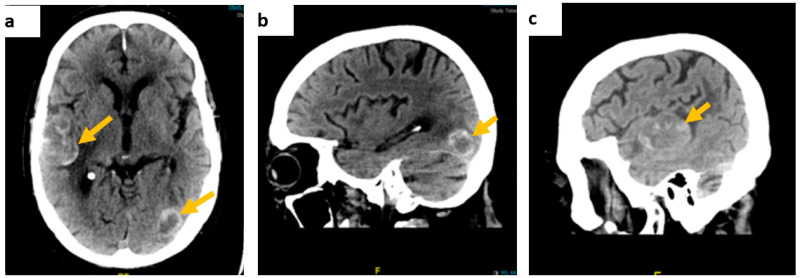
Contrast-enhanced brain CT scan images. (**a**) On axial view, a right temporoparietal lobe lesion measuring approximately 54 × 33 mm and a smaller left temporal lesion, (**b**) on sagittal view, a left occipital lobe lesion, measuring approximately 23 × 18 mm, and (**c**) the right temporal lesion on the sagittal axis.

Contrast-enhanced CT of the brain showed multiple intra-axial dense space-occupying lesions in keeping with haemorrhagic metastases (Figure 1).

**Figure 2:**
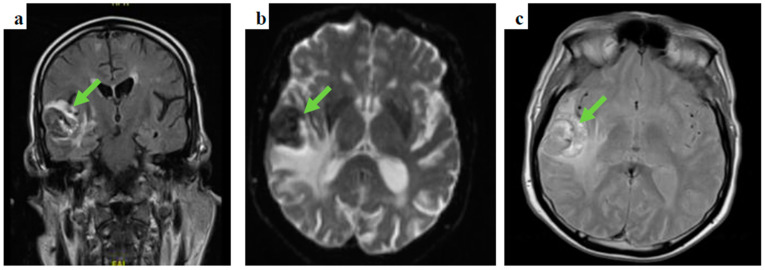
Gadolinium-enhanced brain MRI images: (**a**) coronal, fluid-attenuated inversion recovery (FLAIR) sequence showing a right temporal lobe lesion, (**b**) an axial, diffusion-weighted image (DWI), b1000 showing the temporal lesion surrounded by vasogenic oedema, and (**c**) an axial, T2-weighted imaging showing a high intensity lesion in keeping with the presence of either subacute stage blood products or melanin.

Following prompt discussion with the neuro-surgical team, dexamethasone was added to levetiracetam and the patient underwent gadolinium-enhanced MRI of the brain and contrast-enhanced CT-CAP. The latter did not show evidence of primary malignancy, whereas the gadolinium-enhanced MRI of the brain depicted several space-occupying lesions in the brain parenchyma, the largest measuring 37 × 31 × 28 mm in the right temporal area, in keeping with haemorrhagic brain secondary lesions (Figure 2).

**Figure 3:**
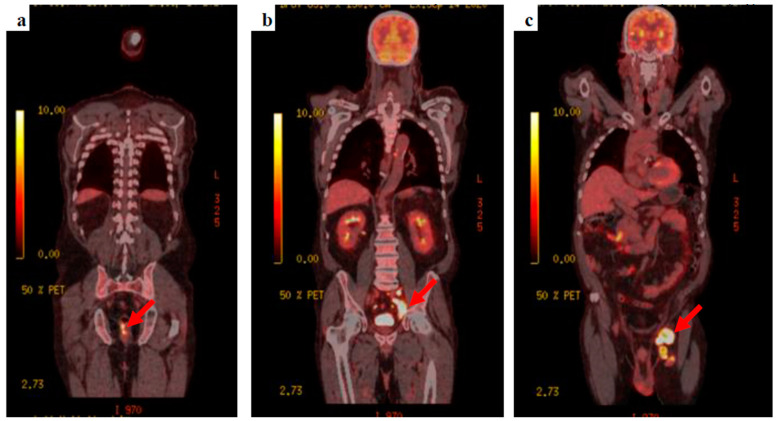
^18^F-FDG PET-CT images (**a**) the anorectal primary lesion with SUV_max_ 12.2, (**b**) deep, left external and common iliac nodal uptake, and (**c**) bulky, extensive, left inguinal lymphadenopathy.

The patient’s clinical condition improved and he was keen to go home, agreeing to be referred to the neuro-oncology multidisciplinary team meeting. Whole-body fluorodeoxyglucose (FDG)-positron emission tomography (PET) was requested to find the primary tumour, showing extensive FDG-avid lymph nodes within the left groin, the left external iliac station, extending into the left common iliac group with retroperitoneal lymphadenopathy extending into the posterior mediastinum. A focus of FDG avid uptake was seen at the 6–9 o’clock position close to the anorectal junction (Figure 3).

Tumour markers were unremarkable (prostate specific antigen (PSA) = 1.0 µg/L and carcinoembryonic antigen (CEA) = 3.2 µg/L). A primary anorectal malignancy with extensive FDG avid nodal metastatic spread was suspected. Following discussion at the lower gastrointestinal multidisciplinary team meeting, a percutaneous ultrasound-guided core biopsy of the left groin node was requested.

**Figure 4:**
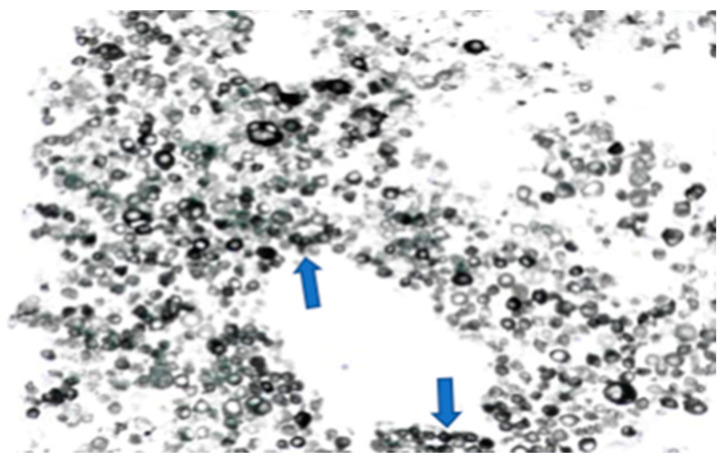
Melanoma cells (numerous dark circle-like cells, melan A staining) on the left groin core biopsy.

The left groin core biopsy showed mostly necrosis with a few surviving cells of metastatic malignant melanoma, with overexpression of S100 and MelanA, but negative for AE1/3, CK18, P63, CD45, and CD30 (Figure 4).

A sample was sent for molecular analysis and the patient referred to the skin multidisciplinary team meeting. There was no history of melanoma, but a history of cutaneous basal cell carcinoma excised in the past was then uncovered.

The neuro-oncology multi-disciplinary team meeting concluded that, due to widespread metastatic cerebral disease, surgical intervention was not feasible and a palliative care referral to a hospice was made. The patient subsequently presented with further seizures and aspiration pneumonia, with contrast-enhanced CT of the brain showing interval increase in size on the previously detected lesions (Figure 1). Following mild clinical improvement, he opted to be discharged home with community palliative care support. Further to this, molecular analysis of the lymph node biopsy showed approximately 60% neoplastic nuclei. No mutations were detected in the regions analysed within the *BRAF* and *KIT* genes however an *NRAS* mutation was detected. At this stage, a decision to start him on an immune checkpoint inhibitor was made with ongoing palliative care support for symptoms.

## 3. Discussion

Melanoma was the 19th most common worldwide primary site of new cancers in both sexes in 2020, with 324,635 cases. Tumour biomarkers are useful in predicting the risk of metastases and thus prognosis [11]. Approximately 3% of melanomas lack an identifiable primary, otherwise known as melanoma of unknown primary (MUP), which is an unusual melanoma subtype that remains biologically ill-defined as compared to the classical melanoma of a known primary [12]. The genomic profiles of patients with cutaneous melanoma and MUP are considerably concordant [13].

This case of metastatic anorectal malignant melanoma reminds us that disseminated solid malignancies may have atypical presentations and that obtaining a histological diagnosis is crucial, and further reinforces why more work needs to be done in this area. As is commonly the case in with such rare conditions, most of the existing literature is based on retrospective case reports and series such as this one.

This patient presented with seizures and the brain MRI findings were consistent with metastatic malignancy. There was no evidence of cutaneous melanoma primarily noted on clinical examination, and the CT-CAP was not able to demonstrate the primary, highlighting some of the limitations of standard imaging techniques. FDG PET findings raised the suspicion of colorectal origin at the anorectal junction and the patient was referred to the colorectal multidisciplinary team meeting based on the most common aetiology for that location and pattern of spread, causing delay in addressing the melanoma primary, with the conclusive diagnosis later revealed by biopsy. This again illustrates the limitations of imaging alone in this condition, and the key role of histology and, particularly, immunohistochemistry, given that a substantial proportion of ARMMs have an amelanotic appearance even microscopically [14].

Checkpoint inhibitors have revolutionized the management of malignant melanoma with drugs like ipilimumab, nivolumab, or pembrolizumab. Some of the pivotal immunotherapy studies such as the CHECKMATE, BMS, and KEYNOTE series of studies, however, either do not set out to include ARMM patients or do not have enough patients in this subgroup or other noncutaneous group of melanomas to infer efficacy. Nevertheless, the use of companion diagnostics and presence of cancer mutational signatures can potentially help to provide hope for use of targeted/immunotherapy in this very rare patient group. Even in instances where there is clearly hope for efficacious therapies, late presentation, burden of spread, and aggressiveness of disease could preclude many patients from taking advantage of these therapies, due to a poor performance status. The patient in this case, was started on Pembrolizumab, a programmed death-ligand 1 (PD-L1) inhibitor but unfortunately, he deteriorated and died before his second cycle.

## 4. Conclusions

This case highlights the diagnostic challenge anorectal melanoma represents, particularly in metastatic presentations. The rarity of this entity in a location where adenocarcinomas from colorectal origin or squamous cell tumours are vastly more common, together with the paucity of literature in the field and the aggressive behaviour of this tumour all contributed to diagnostic delays and poor prognosis to the patient. The biological behaviour of malignant melanocytes in the anorectal area appears to be different to their behaviour in other areas, for reasons not yet clearly understood, which also adds to the need for further research in the area.
